# Cooperative RecA clustering: the key to efficient homology searching

**DOI:** 10.1093/nar/gkx769

**Published:** 2017-08-31

**Authors:** Andrew J. Lee, Rajan Sharma, Jamie K. Hobbs, Christoph Wälti

**Affiliations:** 1 Bioelectronics Group, School of Electronic & Electrical Engineering, University of Leeds, Woodhouse Lane, Leeds, LS2 9JT, UK; 2 Department of Physics and Astronomy, University of Sheffield, Hounsfield Road, Sheffield, S3 7RH, UK; 3 The Krebs Institute, University of Sheffield, Sheffield, S10 2TN, UK

## Abstract

The mechanism by which pre-synaptic RecA nucleoprotein filaments efficiently locate sequence homology across genomic DNA remains unclear. Here, using atomic force microscopy, we directly investigate the intermediates of the RecA-mediated homologous recombination process and find it to be highly cooperative, involving multiple phases. Initially, the process is dominated by a rapid ‘association’ phase, where multiple filaments interact on the same dsDNA simultaneously. This cooperative nature is reconciled by the observation of localized dense clusters of pre-synaptic filaments interacting with the observed dsDNA molecules. This confinement of reactive species within the vicinity of the dsDNA, is likely to play an important role in ensuring that a high interaction rate between the nucleoprotein filaments and the dsDNA can be achieved. This is followed by a slower ‘resolution’ phase, where the synaptic joints either locate sequence homology and progress to a post-synaptic joint, or dissociate from the dsDNA. Surprisingly, the number of simultaneous synaptic joints decreases rapidly after saturation of the dsDNA population, suggesting a reduction in interaction activity of the RecA filaments. We find that the time-scale of this decay is in line with the time-scale of the dispersion of the RecA filament clusters, further emphasising the important role this cooperative phenomena may play in the RecA-facilitated homology search.

## INTRODUCTION

Homologous recombination is critical in maintaining genomic integrity, repairing detrimental DNA damage that would otherwise elicit deleterious cell activity, and effecting genetic diversity during meiosis (1,2). Central to this process is an adenosine triphosphate (ATP)-mediated DNA strand exchange orchestrated by DNA repair proteins—RecA in bacteria, RadA in archaea or Rad51 in eukaryotes—which exchange DNA strands with identical or very similar sequences (3,4). Amongst these, RecA is the most widely studied (5).

**Figure 1.**
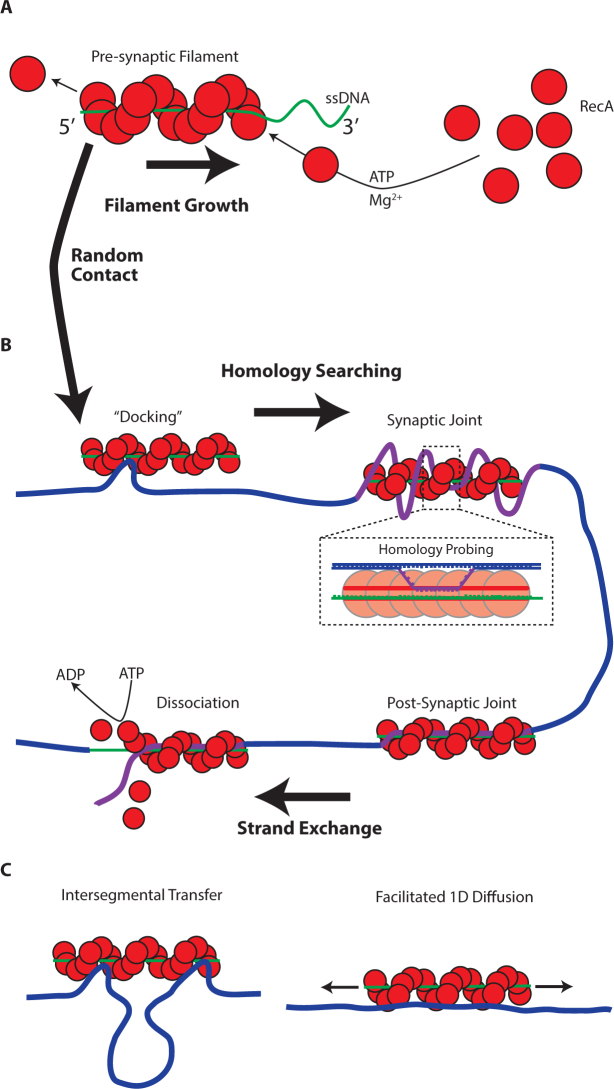
RecA nucleoprotein filament formation and homology searching. (**A**) RecA monomers nucleate and polymerize upon ssDNA in the presence of ATP/ATPγS and Mg^2 +^ to form an active pre-synaptic filament. (**B**) 3D diffusion of filaments results in ‘random contact’ with dsDNA where initial homology probing takes place in a synaptic joint. Successful location of homology results in a three-stranded post-synaptic complex. (**C**) Pre-synaptic filaments can undertake facilitated 1D diffusion along dsDNA and cross between non-contiguous dsDNA regions via intersegmental transfer.

In the presence of ATP or ATPγS (a non-hydrolysable ATP analog) and Mg^2 +^, RecA preferentially binds to single-stranded DNA (ssDNA) at a stoichiometry of 3 nucleotides (nt) per monomer to form a right-handed helical nucleoprotein filament, also known as a pre-synaptic filament (Figure 1A) (6). The RecA proteins are positioned at the outside of the filament and the ssDNA is held along the central axis in the binding site I of RecA, with the nucleotides exposed to the internal cavity (7,8).

The pre-synaptic filament then binds transiently and non-sequence-specifically to one of the strands of an incoming double-stranded DNA (dsDNA) via the weaker binding site II of RecA to form a nascent three-stranded synaptic-joint. In this configuration, both the ssDNA and the dsDNA remain associated with the RecA filament with the latter entering the complex along the helical groove of the filament (8). The binding of the incoming dsDNA into site II induces a kink in the dsDNA, disrupting the base-stacking and considerably weakening the basepairing which leads to a local opening of the dsDNA duplex in the vicinity of the encapsulated ssDNA (8–11). This process has been demonstrated to be enhanced where the stability of the duplex is already compromised (12), and is likely critically dependent upon spontaneous fluctuations in the DNA conformation, commonly referred to as DNA breathing, in line with many sequence specific DNA binding proteins that require transient access to a single strand of the duplex (13,14).

The nucleotides of the locally disrupted incoming dsDNA are able to rotate away from their original pairing position and hydrogen-bond with the available nucleotides of the encapsulated ssDNA to form a transient intermediate (10,15). This complex becomes increasingly stabilized where homology between the encapsulated ssDNA and the incoming dsDNA exists, as an increasing number of basepairs can be formed, and is even capable of overcoming a small degree of heterology. When all relevant basepairs of the incoming dsDNA are broken and new stable base pairing is established between the encapsulated ssDNA of the pre-synaptic filament and its complementary strand on the incoming dsDNA, a strand exchange between the two DNA molecules ensues with the homologous strand of the incoming dsDNA molecule being replaced with the filament-encapsulated ssDNA—transforming the synaptic joint into a stable post-synaptic complex (16,17). At this stage, ATP hydrolysis causes a conformational change in RecA leading to the disassembly of the post-synaptic complex and consequently the release of the bound DNA molecules (18–20). However, where ATPγS is used as the nucleoside cofactor, the post-synaptic complex can remain intact for several hours (Figure 1B) (21).

Although the process of homology sampling appears simple in principle, the underlying mechanism by which a pre-synaptic filament can successfully locate a homologous sequence across genomic DNA within relevant metabolic time-scales, remains poorly understood (5,9,22,23). In particular the sampling method and the nature of the intermediates remain unclear (10,24).

Previous *in vitro* studies proposed a reversible and thermally driven mechanism of random sampling (25,26). More recently, one dimensional facilitated diffusion of the pre-synaptic filament along the contour of the dsDNA molecule was reported where short filaments (≤80 nt) continually searched a 60–300 bp region before dissociating (Figure 1C) (27). This mechanism, while efficient in scanning short dsDNA, however, was ruled out as the dominate searching mechanism for longer dsDNA (several kbp) (28). For longer filaments (≥430 nt), inter-segmental transfer of the pre-synaptic filaments between distal portions of non-contiguous dsDNA (Figure 1C) was observed (29). These studies were restricted to *in vitro* situations and limited to relatively short dsDNA (compared to the size of genomic DNA). Thus the dominant *in vivo* mechanism, which provides a high apparent efficiency, may in fact be a combination of both 1D sliding and inter-segmental transfers, similar to the mechanisms employed by other site specific DNA binding proteins (5).

Recent studies suggested that the process of homologous recombination would be more efficient, and thus realistic on a metabolic time-scale, when a reduction in complexity is achieved by sampling only short tracts of microhomology as small as 6–8 nt in length (30,31).

However, the apparent lack of a detailed understanding of the homology search mechanism, and in particular of the type of intermediates formed during the early stages of homology sampling are, in part, a direct consequence of the employed investigation methodologies, the majority of which have utilized ensemble methods such as biochemical assays (28). More recently, Förster resonance energy transfer (FRET)-based techniques have been employed for studies between short (≤100 bp) pre-synaptic filaments and fully-homologous dsDNA molecules (24,27,32). Although eloquent, none of these methods offer direct observation of the intermediates formed during the homology sampling of synaptic filaments on a longer dsDNA, but merely yield indirect measurements of successful post-synaptic joint formation (27,29,33).

Here, we directly image the intermediate synaptic structures which exists during the RecA-facilitated homology search using atomic force microscopy (AFM) which offers high spatial resolution and the ability to work in both air and physiological buffers. Using a holistic approach, including a combination of direct single molecule static and high-speed dynamic observations with AFM, we demonstrate that a highly cooperative multi-phase interaction landscape exists between the nucleoprotein filaments and the dsDNA during homology sampling. During the initial phase, clusters of pre-synaptic filaments concurrently interact with the same dsDNA, and multiple filaments from the cluster then dock to the dsDNA to form many transient synaptic joints. The majority of the synaptic intermediates dissociate from the dsDNA scaffold in a slower resolution phase, leaving behind a population of only stable post-synaptic complexes where homology is located. In this way, the available sequence space is sampled in a parallel manner thus significantly increasing the overall efficiency.

## MATERIALS AND METHODS

### Materials

RecA protein (*Escherichia coli*) was purchased from New England Biolabs Inc. (NEB; Ipswich, USA) at a concentration of 2 mg/ml in 20 mM Tris–HCl (pH 7.5), 1 mM Dithiothreitol (DTT), 0.1 mM ethylenediaminetetraacetic acid (EDTA), and 5% glycerol, and was used without further purification. Adenosine 5′-(γ-thio)triphosphate (ATPγS) tetralithium salt, which was made up to a final concentration of 5 mM in deionized water; MgAc, made up to 200 mM; NiCl_2_, made up to 10 mM; and Tris-Ac, made up to 300 mM, pH 7.4, were all purchased from Sigma Aldrich (St Louis, USA).

The DNA oligonucleotides used in this study were synthesized by Integrated DNA Technologies (IDT) (Coralville, USA) and purified by desalting. All the dsDNA templates were amplified using PCR from either a modified pGEM-T plasmid vector in the case of the 890 bp fragment (30), or Lambda bacteriophage DNA, in the case of the 3.5 kbp fragment. Lambda bacteriophage DNA was purchased from Sigma Aldrich (St Louis, USA). The nucleotide sequences for all DNA molecules used in this study are provided in the supporting information.

### Single molecule snapshots

#### Formation of RecA nucleoprotein filaments

RecA nucleoprotein filaments were formed on ssDNA oligonucleotides following previously reported protocols (30). Briefly, 1 μl of ssDNA oligonucleotides (10μM) was mixed with RecA protein (at the concentration of one RecA monomer to 3 nt) in the presence of 0.5 mM ATPγS in a buffer containing 10 mM Tris-Ac, 2 mM MgAc (pH 7.4). The reaction was incubated for 15 min at 37°C.

#### Solution snapshots

Nucleoprotein filaments were added to the dsDNA template (in a 3:1 ratio of nucleoprotein filaments:dsDNA template) in a buffer containing 10 mM Tris-Ac, 10 mM MgAc (pH 7.4). The samples were incubated at 37°C between 5 and 60 min as detailed in the text. Reactions were quenched at relevant time points by snap cooling the reaction to 4°C. This approach is validated in the supporting information (Supplementary Figure S1). Samples were then diluted in 10 mM Tris-Ac and 10 mM MgAc (pH 7.4) to the required concentrations, and a total of 10 ng of dsDNA deposited on freshly cleaved mica surfaces which had been pre-incubated with 10 mM NiCl_2_ for 1 min. The mica surfaces were rinsed with deionized water and dried with a stream of dry N_2_.

#### AFM imaging

All samples were imaged in tapping mode (amplitude modulation), in air, with a Bruker Dimension 3100 AFM (Bruker Nanosurfaces, Santa Barbara, USA) using OTESPA etched Si probes. Substrates were randomly sampled to ensure a statistically relevant population of dsDNA molecules were observed, typically *n* ≈ 1000 dsDNA per sampled population. We note, only samples where the termini could be discerned unambiguously, that fell entirely within the imaging area, and that did not overlap with one and other, were analyzed.

### Observation of single molecule dynamics

For observing the *in vitro* dynamics of the RecA-mediated homology search a total of 10 ng of 3.5 kbp dsDNA template was deposited on NiCl_2_ incubated mica in a buffer containing 10 mM Tris-Ac and 10 mM MgAc (pH 7.4) as previously described (34). The mica substrate was washed and subsequently imaged in the same buffer.

Samples were imaged in tapping mode (amplitude modulation), in aqueous buffer, with a Bruker Dimension Fastscan AFM (Bruker Nanosurfaces, Santa Barbara, USA) using Fastscan D etched Si_3_N_4_ cantilevers containing a Si tip. RecA nucleoprotein filaments were formed as described above and introduced during imaging at a 1:1 ratio of RecA filaments to dsDNA template. The homology searching interaction was followed at 22°C by continuous AFM scanning.

## RESULTS AND DISCUSSIONS

**Figure 2.**
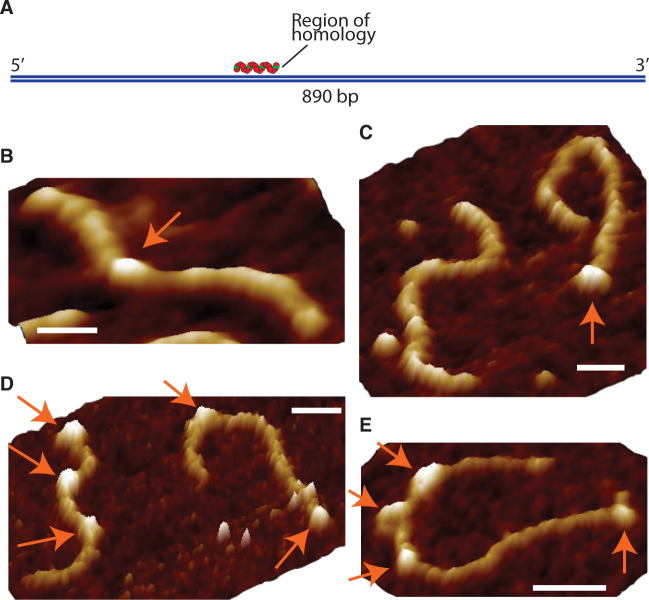
Parallel RecA homology searching. (**A**) Schematic depicting the homologous region on the 890 bp dsDNA for the 60-nt-long RecA nucleoprotein filaments used in this study. Panels (**B–E**) show a selection of reaction intermediates, with: (B) a successfully formed post-synaptic complex at the homologous sequence; (C) free filaments and dsDNA alongside a synaptic joint bound at the terminal of an 890 bp dsDNA; and (D and E) multiple synaptic joints formed along dsDNA molecules. Nucleoprotein filaments are highlighted with arrows. All scale bars = 60 nm, all z scales = 8 nm.

In order to study the intermediates formed between pre-synaptic filaments and dsDNA during the RecA-facilitated homology search, AFM images of the reaction were acquired periodically over the time-course of an hour. For this, nucleoprotein filaments formed on 60-nt-long ssDNA were incubated with 890 bp dsDNA containing a 60 bp region of homology. The region of homology was located 360 bp away from the 5′ terminal, i.e. 85 bp away from the centre, to enable clear distinction between synaptic and the post-synaptic joints (see Figure 2A). 60-nt-long nucleoprotein filaments have been shown to be highly efficient at forming synaptic joints(30,35) and are of comparable length to those used in previous FRET-based studies (24).

The successful formation of post-synaptic complexes at the correct location was independently validated using a restriction enzyme assay (Supplementary Figure S2) (30,35). The 890-bp dsDNA was designed to contain an BanI restriction site within the region of homology, and hence the formation of post-synaptic complexes prevented access of the restriction enzyme and thus digestion of the 890 bp into smaller fragments. The 890-bp DNA was however digested into short fragments when either a non-homologous ssDNA or no filament was formed. The results are shown in Supplementary Figure S2.

For the initial AFM study, the homology searching reaction was quenched at various time points between 5 and 60 min by rapidly cooling the sample to 4°C. To characterize the different intermediates which exist during the initial stages of RecA-facilitated homologous recombination, the samples were investigated by AFM and the number and type of intermediates formed between the dsDNA and the nucleoprotein filaments were evaluated. The dsDNA were categorized according to the observed interaction state, i.e. those which: (i) did not interact with any nucleoprotein filaments; (ii) featured one or more synaptic joints; and (iii) those dsDNA substrates that featured a singular nucleoprotein filament attached at the region of sequence homology. We note that only those DNA species containing no other nucleoprotein filament interactions were considered to have resolved into a final post-synaptic complex in our study.

It is important to note that for a reaction kept at 4°C for 30 min—the typical time lapse between quenching the reaction and AFM image acquisition—<1% of the dsDNA were found to contain synaptic joints, and indeed even after 60 min incubation of the reaction at 4°C, only 6% of the dsDNA were found to have formed intermediates at any point along the molecule (Supplementary Figure S1B).

### Rapid association phase

The challenges in capturing the rapid initial stages of association of the pre-synaptic filaments and the incoming dsDNA before stable synaptic and post-synaptic joints are formed has been highlighted in previous studies. (10,24) However, these studies were limited by either spatial resolution and/or the inability to distinguish between multiple simultaneous events. Here, we employ AFM to provide the spatial resolution required to distinguish between individual events. An AFM image of a representative example of a post-synaptic complex can be seen in Figure 2B, and synaptic complexes present at heterologous locations on the dsDNA can be seen in Figure 2C–E.

**Figure 3.**
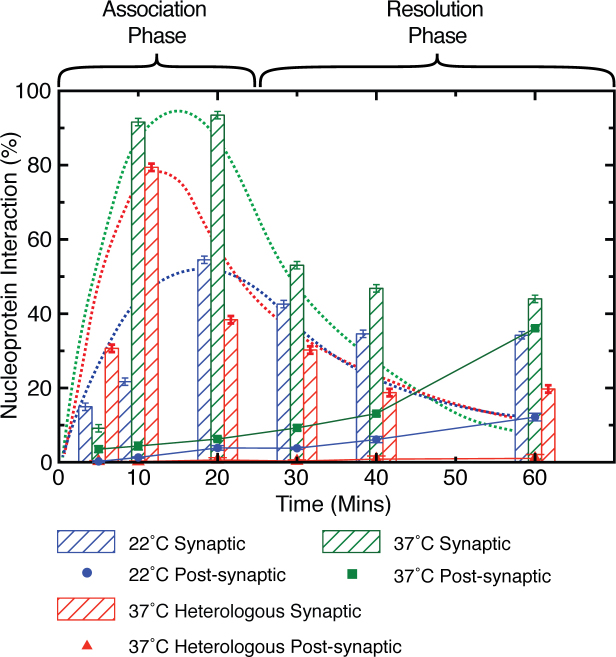
Homology searching. Histogram depicting the percentage of dsDNA featuring one or more synaptic joints or post-synaptic joints at 37°C (green bars) and 22°C degrees (blue bars), respectively, as a function of reaction time. Clearly, the reaction is biphasic, featuring a rapid increase (association phase) followed by a slower decrease (resolution phase). The dashed lines are a guide to the eye only, indicating the number of dsDNA featuring one or more synaptic joints but not singular post-synaptic joints. The number of post-synaptic joints is shown by solid symbols (green squares and blue circles, respectively), which increases steadily over the time-frame of these experiments. The same biphasic behaviour is observed for heterologous nucleoprotein filaments (red bars), but without the increase in the number of post-synaptic joints (red triangles). The solid lines serve as a guide to the eye.

The percentage of dsDNA containing synaptic joints is shown as a function of time in the histograms of Figure 3. For a RecA-facilitated homologous recombination reaction carried out at 37°C, we found that the percentage of the dsDNA population which features one or more synaptic or post-synaptic joints increased very rapidly and reached a maximum at around 92 ± 1% within the first 10 min of the reaction (green histogram). Of this, only a small proportion, 4.4 ± 1%, featured a singular post-synaptic joint formed at the correct homologous location (solid green squares), while the remaining 87 ± 1% had one or more synaptic joints formed at heterologous locations.

On average we observed about three synaptic joints per dsDNA molecule during the association phase (Supplementary Figure S3), which corresponds to an average of over 20% of the dsDNA being concurrently probed for homology. This clearly demonstrates that the homology search begins in a highly parallel fashion. This is contrary to previous studies, which interpreted the multiple contacts between the dsDNA and RecA-based nucleoprotein filaments as inter-segmental transfer based on the interactions between a single nucleoprotein filament and the dsDNA substrate (29). Arguably, multiplexing of the homology search increases the efficiency of the process, and, when extrapolated to genomic DNA, makes a genome-wide search a much more feasible prospect. Indeed, it could be argued that parallelization is a prerequisite for any searching mechanism that is entirely thermally driven. However, as expected, the chances of an individual filament locating the region of homology at the first encounter remains low even for the relatively short 890-bp DNA molecule utilized here.

A similar pattern was observed when the RecA-mediated homologous recombination reaction was carried out at 22°C (blue histogram in Figure 3) rather than 37°C. However, saturation is only reached after about 20 min and only at a significantly lower level. In this case, 55 ± 4% of the dsDNA population were found to feature one or more reaction intermediates, i.e. synaptic or post-synaptic joints, of which only 3.6 ± 0.6% were found to be singular post-synaptic joints (solid blue circles in figure 3). The observed temperature dependence is in line with the previously reported indications that the searching mechanism is thermally driven (1,18), although whether the reduced maximum and the slower timescale are a direct consequence of the reduced diffusion of either the nucleoprotein filament or the dsDNA into the complex, or the reduction in local DNA breathing, is unclear, and most likely the observed behaviour is a combination of the different effects (36).

### Resolution phase

Surprisingly, the initial rapid increase of dsDNA–nucleoprotein filament intermediates is followed by a second phase, which, when the reaction is carried out at 37°C, starts from about 10–20 min. In this phase, the population of dsDNA displaying a reaction intermediate rapidly decreased to around 43 ± 1% at the 60-min-timepoint (Figure 3). This is different to the current understanding of RecA-facilitated homologous recombination *in vitro*. In this case, the nucleoprotein filaments are thought to form a synaptic joint with a dsDNA which then stays associated with the dsDNA for a finite amount of time and probes the vicinity of the initial interaction point for potential sequence homology. Unless a post-synaptic joint is established, the filament is likely to disassociate from the dsDNA within minutes (32). The rate of hydrolysis of the nucleoside co-factor ATPγS is of the order of 0.01 min^−1^ (37), and hence the stability of the RecA nucleoprotein filaments formed with ATPγS exceeds the typical timescale of the experiments discussed here. It would thus be reasonable to assume that, within the timescale of this experiment, any nucleoprotein filament dissociating from dsDNA to be fully intact and active, and hence able to interact with a new dsDNA in the same way again. Over the course of this experiment, the concentration of available nucleoprotein filaments will decrease by <15% given that the starting ratio of filaments to dsDNA is 3:1, and that no more than 40% of the dsDNA will establish a post-synaptic joint over the timescale of this experiment (see below).

Taken together, these facts would imply that no significant decrease in the number of dsDNA featuring one or more synaptic or post-synaptic joints would occur over the time-scale of this experiment, akin to typical binding kinetics curves. This is clearly not bourne out by the data shown in Figure 3, which thus suggests that the interaction rate of the nucleoprotein filaments with the dsDNA decreases very rapidly. We note that a similar decay would be observed if the dissociation rate increased over time. However, if anything, the opposite is likely to be the case and the apparent average dissociation rate may be expected to increase as some synaptic joints incur micro–homology and thus become stabilized. Therefore, the timescale of the reduction in interaction activity is likely to be faster than the timescale of the observed decay in the number of dsDNA featuring one or more synaptic filaments.

The same behaviour was observed when employing non-homologous 60-nt-long filaments. A similar number of synaptic joints was seen to form during the early rapid association phase of the homologous recombination reaction, followed by a rapid decrease during the second phase (red histogram in Figure 3). Furthermore, the number of interaction intermediates reaches zero after an overnight incubation, resulting in bare dsDNA substrates as the RecA monomers dissociates from the filament following hydrolysis of the nucleoside co-factor ATPγS (1) (at a rate of 0.01 min^−1^ (37)).

In contrast, the percentage of dsDNA featuring a nucleoprotein filament singularly occupying the region of homology increases steadily over the same period to around 36±1% for the reaction carried out at 37°C. This increase is expected and is likely resulting from the formation of a stable three-stranded post-synaptic joint when one of the synaptic intermediates successfully locates the homologous site, while all the other relatively weakly interacting heterologous synaptic joints dissociate from the dsDNA. We note that the number of dsDNA featuring a post-synaptic joint, but no synaptic joints, is increasing steadily over the investigated time period of 60 min, and no new synaptic joints are formed on these dsDNA. This confirms our earlier conclusion that the initially relatively high interaction rate of the nucleoprotein filaments with the dsDNA decreases very rapidly, and effectively reaches zero after only 10s of min. For the heterologous nucleoprotein filaments, no progressions to post-synaptic joints were observed as expected (red solid triangles in Figure 3).

Overall, the results reported in Figure 3 suggest that the RecA-facilitated homology search mechanism is multi-phased, initially dominated by a rapid highly parallel ‘association’ phase, followed by a slower ‘resolution’ phase. This is in line with previous reports of the transient nature of the initial contact between the nucleoprotein filament and the dsDNA (11).

Where new base pairs are formed between the filament-encapsulated ssDNA and an incoming dsDNA strand the initially weak interaction is stablized processively at regions of sequence homology (10,38). However in the absence of homology, the interaction remains transient and is potentially able to undertake facilitated diffusion along the dsDNA to another location (27) or disassociate from the substrate entirely.

After the initial fast association phase, in all cases, a significant reduction in the number of dsDNA featuring one or more synaptic or post-synaptic joints is observed. This implies that the number of dsDNA not interacting with any RecA nucleoprotein filaments increases steadily after about 10–20 min. It is well established that the nucleoside co-factor employed in these studies, ATPγS, hydrolysis at a rate of 0.01 min^−1^ (37), which corresponds to an expected decay time of the nucleoprotein filaments of around 2 h. However, the observed time-scale of the decrease in dsDNA featuring synaptic joints is significantly faster. In the case of the heterologous nucleoprotein filament, the observed decay is not complicated by the simultaneous formation of post-synaptic joints, and thus provides a good model to study the decay in isolation. The observed reduction follows an exponential decay with a decay time of 580 s, i.e. ∼10 min (see Supplementary Figure S4). We note that decay times of 10 min are also compatible with both homologous cases (37°C and 22°C). However, these timescales are an order of magnitude faster than the ones imposed by the hydrolysis of the nucleoside co-factor, therefore suggesting that another, additional, mechanism governs the efficiency of RecA facilitated homologous recombination.

The fact that the synaptic joints are short-lived unless homology can be established (32) is in line with our observation of the rapid decrease of dsDNA featuring synaptic joints (resolution phase). It has been argued that this short-livedness enables each filament to interrogate a large number of dsDNA in quick succession, thereby probing a large volume of sequence space very rapidly and efficiently. However, the results reported above suggest that the ability of the nucleoprotein filaments to interact with dsDNA decreases very rapidly, on a time-scale of minutes, and consequently suggest that the initial efficiency during the association phase is high enough to allow genome-wide homology probing. The fact that multiple filaments interact simultaneously with each dsDNA is likely a key factor in ensuring this high initial activity.

We note that to date, the impact of the nucleoprotein filament length on the efficiency of the homology search is largely unknown. It has been reported that as little as 6–8 nt of sequence homology is sufficient to stabilize a dsDNA–nucleoprotein filament post-synaptic joint (30,31,39); however this has typically been associated with poor efficiencies. Longer pre-synaptic joints can potentially form multiple initial synaptic joints simultaneously (10), and hence longer filaments may be able to probe multiple points in the sequence space concurrently. As a result, the average residence time of a nucleoprotein filament on the dsDNA substrate is expected be longer due to the additive effect of the multiple weak contacts (27,32). In addition, larger filaments exhibit a reduced diffusion constant which could further impact on the efficiency of the overall homology search.

### Homologous hunting in local packs—a highly efficient cooperative process

In order to investigate further the apparent cooperative nature of the RecA-facilitated homology search, we used high-speed AFM (HS-AFM) to observe the interaction dynamics between nucleoprotein filaments and dsDNA in real-time and with true single molecule resolution. In contrast to most FRET and biochemical assays, HS-AFM offers sufficient spatio-temporal resolution to enable true and direct single molecule investigations. For this set of experiments, 3.5 kbp DNA molecules were partially adhered to a mica surface following the protocol described elsewhere (34). Longer dsDNA benefit from better surface adsorption to resist the impact of the scanning probe whilst retaining multiple degrees of translational freedom, making a 3.5 kbp dsDNA more appropriate for these experiments than the 890 bp DNA used above (34). 60-nt-long nucleoprotein filaments were introduced to the transiently surface-bound DNA and the interactions were followed by continuously scanning the sample with HS-AFM.

**Figure 4.**
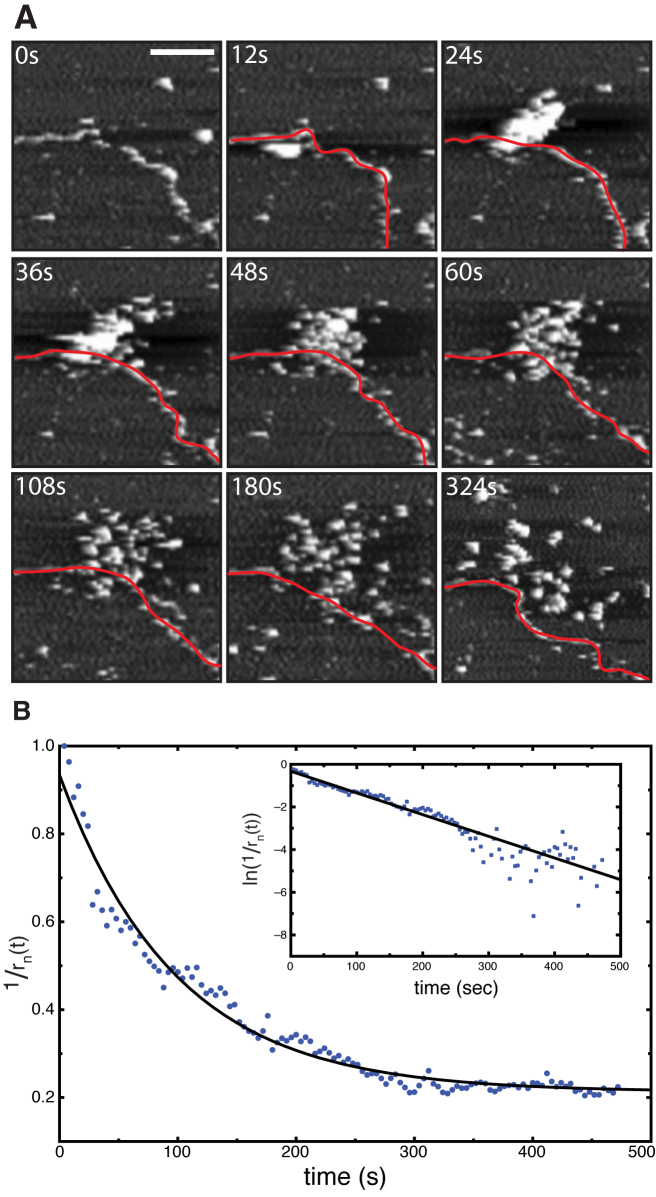
Parallel homology probing orchestrated by cooperative clusters of pre-synaptic RecA filaments. (**A**) A series of high speed AFM images depicting the arrival of a cluster of 60-nt-long RecA nucleoprotein filaments and their subsequent interaction with a 3.5 kbp dsDNA molecule loosely bound to a mica substrate. The dsDNA is highlighted in red as a guide to the eye. (**B**) Plot showing the inverse of the normalized radius of a representative filament cluster versus time. The solid line represents a fit of an exponential decay to the data. Scale bar = 50 nm, z scale = 6 nm.

Figure 4A (and Supplementary Movie S1) show a representative series of frames depicting the interaction of RecA-based nucleoprotein filaments with surface-tethered dsDNA. The arrival of a large number of nucleoprotein filaments and their subsequent interaction with the dsDNA can be seen clearly. Surprisingly, the arriving filaments were not uniformly distributed, but instead appeared in tightly packed localized clusters (Figure 4A, 30 s). Upon arrival on the surface, the individual nucleoprotein complexes were found to be able to diffuse freely, not noticeably constrained by the other filaments in the cluster, suggesting that the clusters represent cooperative arrangements rather than non-specific aggregation (Figure 4A, 30 s — 228 s). From these clusters, multiple nucleoprotein filaments can be seen to interact simultaneously with the same dsDNA, in line with our observation of parallel reaction intermediates during the initial rapid association phase. Evidence of such nucleoprotein filament clusters can be seen across all time points observed in this study, however it is noted that their prevalence decreases significantly with time (see Supplementary Figure S5).

The formation of clusters locally enhances the nucleoprotein filament concentration around the dsDNA, and it is reasonable to assume that the number of synaptic joints formed on a particular dsDNA upon interaction with a single cluster depends on the density and hence the size of the cluster at the time of the interaction—the further spread out the cluster is, the less likely it is for a particular pre-synaptic filament in the cluster to bind to the dsDNA to form a synaptic joint. In fact, the decay of the dsDNA featuring a synaptic joint observed during the resolution phase might be related to the dispersion of these clusters and thus the decrease of the locally enhanced concentrations.

From Figure 4A it can be seen that the nucleoprotein filament cluster rapidly disperses over time. As a first order naïve approximation, we can assume the interaction probability of the nucleoprotein filament with the dsDNA to be inversely proportional to the average linear dimension of the cluster, and thus the reaction efficiency can be estimated from the time course of the cluster size.

Figure 4B shows the normalized inverse of the average cluster radius as a function of time for a representative cluster. The time point *t* = 0 was chosen as the first frame where the cluster was observed. The cluster dispersion follows an exponential behaviour, and hence an exponential was fitted to the data in figure 4B, which yielded a decay time of ∼94 s or 1.5 min (solid line in figure 4B).

In contrast to the typical time-scale of ATPγS hydrolysis, the observed time-scale for the cluster dispersion is significantly faster (∼6×) than the typical decay time observed for the synaptic joint formation ∼1.5 min versus 10 min versus 120 min). We note that the observed dispersion of the clusters took place while the clusters were in close proximity of the mica surface and this, together with the fact that they were exposed to a continuous force imposed by the scanning AFM tip, may have accelerated the decay of the only very weakly bound-together clusters. In addition, the experiments discussed in the first part of this work were carried out in solution rather than on surface and hence may not be directly comparable. Thus the measured decay rate for the on-surface clusters should only be regarded as an upper limit for the dispersion of clusters in solution. Furthermore, the observed decrease in synaptic joint formation is a convolution of multiple effects, including the dispersion of the cluster as well as the increase of synaptic joints due to the rapid association phase. Hence, the true decay rate of the interaction efficiency is likely to be faster, further reducing the observed discrepancy between the effective decay rate of the efficiency of homologous recombination and the dispersion of the nucleoprotein clusters.

The observation that RecA-based pre-synaptic filaments form cluster is not entirely unexpected. Previously, RecA-based nucleoprotein filaments have been shown to associate with one and other, and in some cases, form bundles and aggregates (40). These bundles have been shown to form *in vivo* in bacteria and it was suggested that they accelerate homology searching (41), supporting the hypothesis that the clusters observed in this study serve a similar purpose. However, here the nucleoprotein filaments in the clusters were only observed to interact with the dsDNA molecule individually, in contrast to the filament bundles which interacted as a whole. Furthermore, bundle formation has generally been reported for nucleoprotein filaments formed with long ssDNA (40,41), and such filamentous bundles have recently been shown to mediate homology pairing of two distant sister DNA (41). Hence, the two processes are likely to be distinct and play unique roles in the process of homologous recombination. However, we note that previous studies have demonstrated that the region responsible for bundle formation overlaps significantly with the secondary DNA-binding site (F270–K322) located within the C-terminal domain, but that the conformation of this region differs considerably between the free protein and the protein in the bundle (42). This suggests that bundle formation and homology searching may be mutually exclusive. Given that the previously reported bundle formation and the cluster formation observed here may be related, the dispersion of RecA clusters may depend on the presence of dsDNA.

In any case, the fact that the nucleoprotein filaments occur in cooperative clusters at the point of interaction with the dsDNA must play an important role in *in vitro* studies of the homology searching mechanisms. Such clusters are very likely to exist in many of the homologous recombination studies conducted to date—and in fact might be a necessary prerequisite for the process to be efficient enough to be observed—but, the existence of clusters is not generally considered in discussions and interpretation of the kinetics or proposed mechanisms of homologous recombination. However, we note that the experimental conditions of this *in vitro* study differ from typical *in vivo* conditions in that the length of the filament is significantly shorter than those generally found *in vivo*. Hence, multiple concurrent interactions of single filaments with different regions of the dsDNA—which play an important role *in vivo—*are absent under the conditions employed here, suggesting that the impact of the observed cluster formation is likely to differ between *in vitro* and *in vivo* experiments.

## CONCLUSION

This work directly interrogates the different stages of the RecA-facilitated homology search under the conditions utilized for previous single molecule experiments, i.e. the process by which RecA nucleoprotein filaments associate with dsDNA, search for and locate sequence homology, potentially amongst vast stretches of heterology. Here, AFM has been employed to assess the different types of intermediates formed during the homology search across a 60-min reaction window. We found that the interaction landscape is considerably more complex than has been suggested by indirect single molecule studies to date; however, previously reported studies were limited by either spatial resolution and/or the ability to distinguish between multiple events. In this study, we showed that the RecA protein-mediated homology search occurs in multiple, distinct stages with different relevant timescales.

RecA-based nucleoprotein filaments were shown to interact with dsDNA in a highly cooperative manner during a rapid ‘association’ phase, where multiple synaptic joints are formed per dsDNA molecule. We observed that on average over 20% of the available sequence space was simultaneously occupied by active RecA nucleoprotein filaments. This highly parallel search reduces the time required to probe the available sequence space significantly.

The association phase is followed by a second phase, the ‘resolution’ phase, where only the post-synaptic joints, i.e. the filaments which successfully located a site of sequence homology, remain bound to the DNA molecule, while the only weakly interacting synaptic joints which were unable to locate sequence homology, dissociate. The number of post-synaptic complexes was found to increase steadily over the course of the reaction to reach almost 40% after 60 min, in line with previous studies on similar RecA-based systems. In contrast, the number of dsDNA featuring one or more synaptic joints was found to decrease rapidly after the initial association phase, suggesting that the ability of pre-synaptic joints to interact with dsDNA is very high at the start of the reaction but decreases rapidly within minutes.

The occurrence of multiple synaptic joints per dsDNA during the initial rapid association phase is reconciled by the observation of dense RecA nucleoprotein clusters, which were resolved in real-time by dynamic AFM studies. These clusters seem to increase the local filament concentration in the vicinity of the dsDNA molecules, thus facilitating a more efficient interaction through the confinement of reactive species. We propose that the formation of such clusters through cooperative arrangements plays an important part in enabling an interaction rate between the pre-synaptic filaments and dsDNA high enough to make a genome-wide homologous search possible on a relevant metabolic timescale.

In summary, we have demonstrated the application of a true single molecule technique—AFM—to provide useful insight into the complex mechanism of homology search by RecA nucleoprotein filaments. The observations discussed here have wide implications on the current understanding of the RecA-mediated homology search as derived from *in vitro* indirect single molecule studies to date, due to these previously unappreciated factors.

## DATA AVAILABILITY

Data supporting this work can be accessed via the University of Leeds repository: https://doi.org/10.5518/243.

## Supplementary Material

Supplementary Data
