# Supplementary material for: Cooperative RecA clustering: the key to efficient homology searching
Source: Nucleic Acids Res. 2017 Aug 31;45(20):11743–51. doi: 10.1093/nar/gkx769 (PMC5714135; doi:10.1093/nar/gkx769)
Supplement: Supplementary Data [file gkx769_supp.zip › nar-00817-f-2017-File008.pdf]

Published online XX-XX-XXXX *Nucleic Acids Research, XXXX, Vol. XX, No. XX* **1–1**
doi:10.1093/nar/gkn000

Cooperative RecA Clustering: The Key to Efficient Homology Searching

Andrew J. Lee [1,2] *, Rajan Sharma [1], Jamie K. Hobbs [2,3] & Christoph Wälti [1] †

[1]Bioelectronics Group, School of Electronic & Electrical Engineering, University of Leeds, Woodhouse Lane, Leeds, LS2 9JT, UK
[2]Department of Physics and Astronomy, University of Sheffield, Hounsfield Road, Sheffield, S3 7RH, UK
[3]The Krebs Institute, University of Sheffield, Sheffield, S10 2TN, UK

Received XXXX; Revised XXXX; Accepted XXXX

SUPPORTING INFORMATION

Validation of quenching method

Figure S1 shows the validation of the quenching approach used to cease the nucleoprotein interactions at regular time intervals of 5 – 60 minutes. Quenching at 4°C was found to be sufficient for ceasing any interactions between the nucleoprotein filaments and the dsDNA scaffold (Figure S1), with only 6.4 % of the surface bound DNA population indicated the formation of a synaptic – and no post-synaptic – complexes at 60 minutes compared to approximately 40 % at 37°C. From figure S1 it can be seen that the majority of the nucleoprotein filaments are present within the background when incubated at 4°C, indicating limited – if any – activity.

Validation of the nucleoprotein interaction with dsDNA

Nucleoprotein filaments were formed on 60 nt oligonucleotides in accordance with the stated methods. The resultant nucleoprotein filaments were incubated with 890 bp dsDNA at a 3:1 ratio for 1 hour. Subsequently, all DNA species were digested with BanI for 30 minutes, followed by complete protein removal by Proteinase K incubation. In the case of successful digestion, the 890 bp dsDNA is cleaved into two fragments of different length (540 bp and 350 bp).

The results of the restriction digest assay are shown in Figure S2, where clear protection of the 890 bp template is observed as a result of RecA post-synaptic joint formation (lane indicated by ’3’). In contrast, for the cases where essential components for the formation of active RecA nucleoprotein filaments were omitted (ssDNA (lanes indicated by ’1’) and RecA (lanes indicated by ’2’)), or where a heterologous 60 nt oligonucleotide is utilised, then no post-synaptic joints were formed and therefore no protection against restriction digestion was observed.

Number of nucleoprotein filaments per 890 bp template

Figure S3 shows the frequency distributions of the number of bound nucleoprotein filaments per DNA template at the 10, 30 and 60 minute time points. A clear shift from multiple filaments interacting in parallel — with an average of about 3 and 2 per DNA template at 10 and 30 minutes, respectively — to predominately singularly-bound nucleoprotein filaments — representing predominately post-synaptic joints — at 60 minutes, can be observed.

Resolution phase decay rate

During the resolution phase, the number of dsDNA featuring one or more synaptic joint decreases rapidly. Figure S4 shows the number of dsDNA featuring one or more synaptic joints in the case of a heterologous RecA nucleoprotein filament. The solid line indicates a least-square fit of an exponential decay, yielding a decay time of about 580 s.

Prevalence of nucleoprotein filament clusters

The prevalence of nucleoprotein filament clusters is found to decrease over the course of a 60 minute reaction. Figure S5 shows a series of AFM pictures taken at different time-points during a 60 minute reaction. The number of the dense clusters diminishes during the resolution phase (20 – 60 minutes) and the clusters are observed to disperse giving rise to a more homogeneous concentration.

Hunting in local packs: HS-AFM study

Movie S1 shows the arrival of a cluster of RecA nucleoprotein filaments and the subsequent interaction of the filaments with transiently surface-bound dsDNA. Movie S1 is presented in support of Figure 4.

Comparison of dsDNA and nucleoprotein filament substrates

A clear difference can be seen between the dsDNA (purple arrows) and bound 60 nt nucleoprotein filaments (green arrows) in this study (figure S6, A - C) when compared to fully polymerised RecA on dsDNA forming long nucleoprotein filaments (figure S6, D - F, yellow arrows). Momomeric RecA in the background is highlighted (white arrows).

*To whom correspondence should be addressed. Tel: +44 1133432266; Email: A.Lee@Leeds.ac.uk
†To whom correspondence should be addressed. Tel: +44 1133432023; Email: C.Walti@Leeds.ac.uk

© XXXX The Author(s)
This is an Open Access article distributed under the terms of the Creative Commons Attribution Non-Commercial License (http://creativecommons.org/licenses/by-nc/2.0/uk/) which permits unrestricted non-commercial use, distribution, and reproduction in any medium, provided the original work is properly cited.

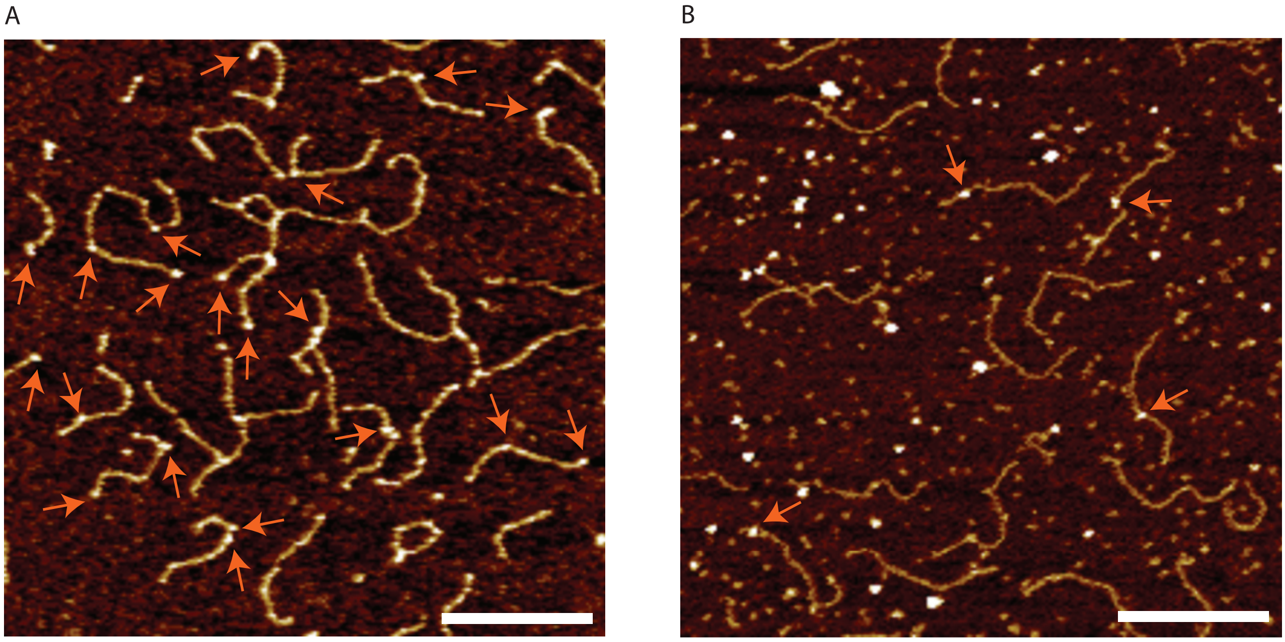

Figure S1. A comparison of patterning efficiencies at 37°C and 4°C. Representative examples of 60-nt-long nucleoprotein filaments (indicated by orange arrows) are shown to interact with 890 bp at 37°C **(A)** and to an extremely limited degree only at 4°C **(B)**, where the majority of NPFs are observed in the background. Scale bars = 250 nm, z scale = 8 nm.

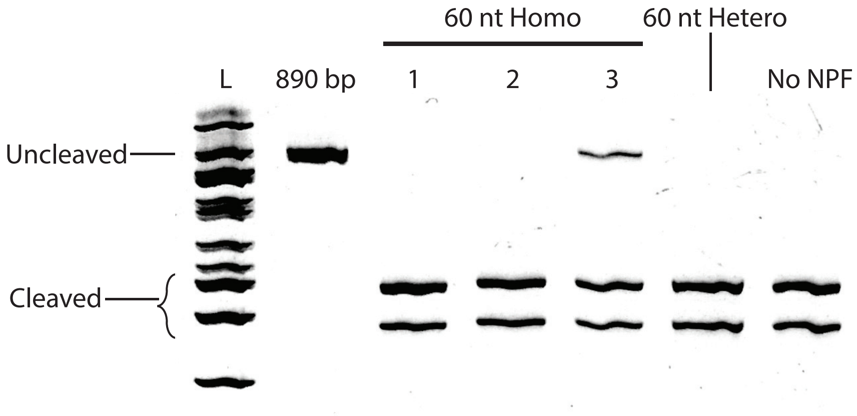

Figure S2. RecA restriction digest gel of 890 bp. A 8% polyacrylamide gel depicting the restriction assay of an 890 bp triple–stranded complex formed with 60 nt homologous and heterologous nucleoprotein filaments at a 3 to 1 ratio. 1 = No oligonucleotide, 2 = No RecA, 3 = Complete nucleoprotein filament.

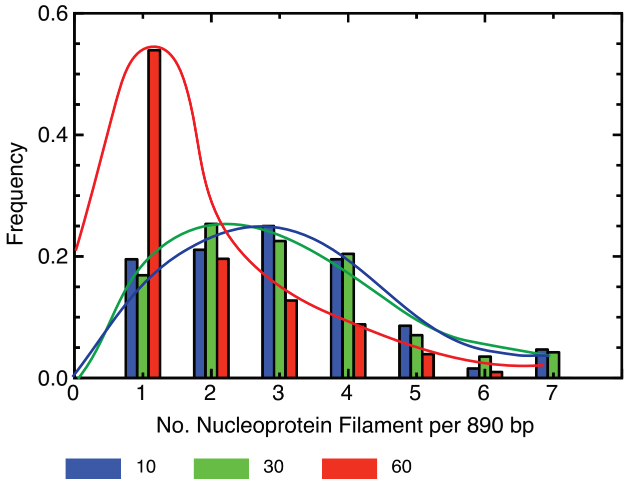

Figure S3. Number of nucleoprotein filaments per 890bp template. A histogram depicting the number of 60-nt-long nucleoprotein filaments per 890 bp template at different time-points (blue: 10 minutes, green: 30 minutes, red: 60 minutes). The solid lines are intended as a guide to the eye only.

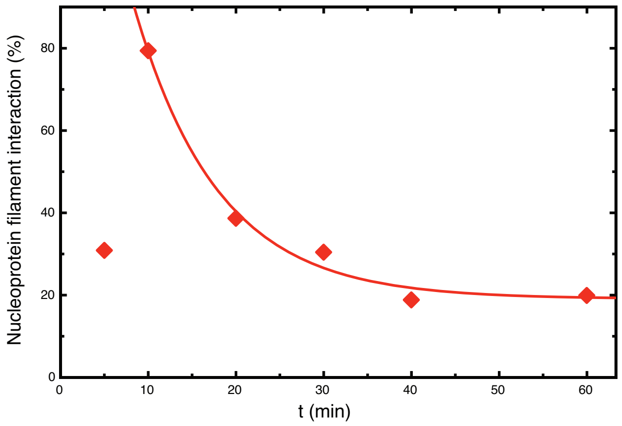

Figure S4. Reduction of synaptic joints during resolution phase Number of dsDNA featuring one or more synaptic joints formed from heterologous RecA nucleoprotein filaments. The solid line indicates the exponential decay with a time-constant of about 580 s.

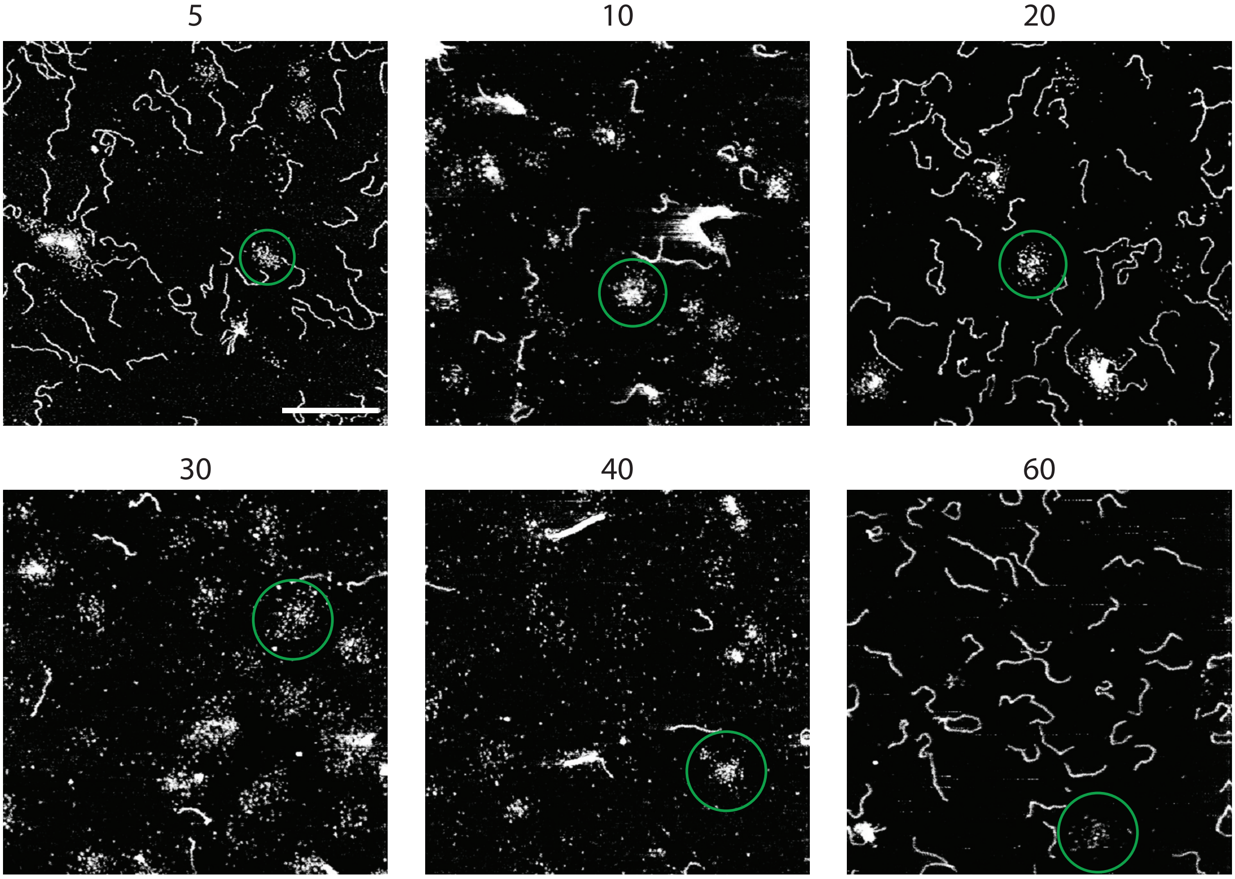

Figure S5. Parallel homology probing orchestrated by cooperative clusters Evidence for the existence of nucleoprotein filament clusters can be observed in static AFM images captured over a 60-minute reaction time. Examples of clusters are highlighted by green circles. Scale bars = 500 nm, z scales = 6 nm.

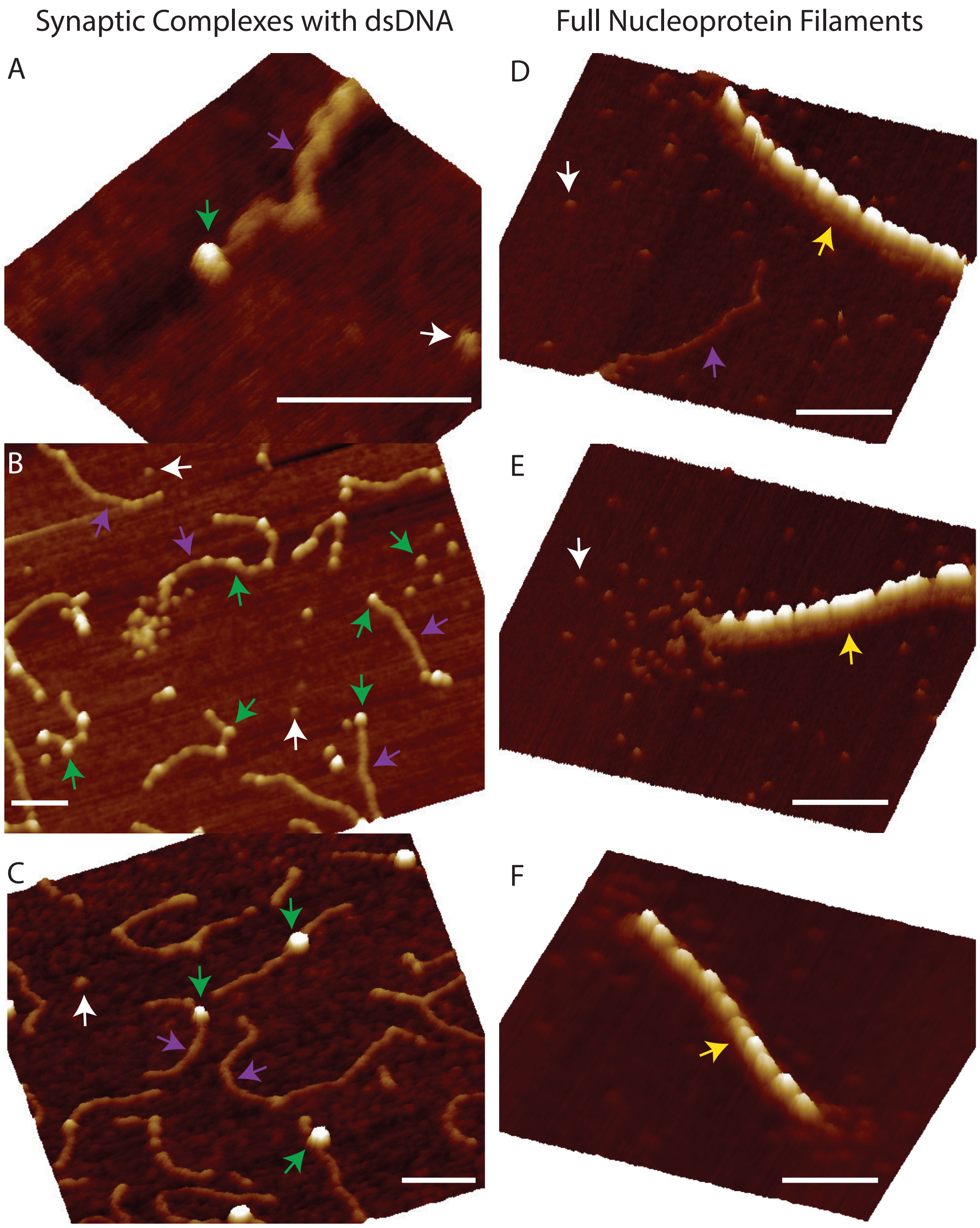

Figure S6. Comparison of the dsDNA and nucleoprotein filament substrates. (A – C) dsDNA (purple arrows) with 60 nt nucleoprotein filaments (green arrows) bound, demonstrating that bare dDNA and dsDNA with a RecA-nucleoprotein filament bound can clearly be distinguished. (D - F) dsDNA fully encapsulated in RecA (yellow arrows) alongside dsDNA (purple arrows) and monomeric RecA (white arrows). Scale bars = 100 nm, Z scales = 6 nm.

890 bp Substrate

60 nt Oligo - Homologous

GATGGCCCACTACGTGAACCATCACCCTAATCAAGTTTTTTGGGGTCGAGGTGCCGTAAA

60 nt Oligo - Heterologous

TATGGTGTTGTCTGTTATGTTCTGTATCTATCATTGAGTCATCTTAGCTACAGTAGTACG

890 bp

GGGGTTCCGCGCACATTTCCCCGAAAAGTGCCACCTGATGCGGTGTGAAATACCGCACAGATGCG
TAAGGAGAAAATACCGCATCAGGAAATTGTAAGCGTTAATATTTTGTTAAAATTCGCGTTAAATTT
TTGTTAAATCAGCTCATTTTTTAACCAATAGGCCGAAATCGGCAAAATCCCTTATAAATCAAAAGA
ATAGACCGAGATAGGGTTGAGTGTTGTTCCAGTTTGGAACAAGAGTCCACTATTAAAGAACGTGG
ACTCCAACGTCAAAGGGCGAAAAACCGTCTATCAGGGCGATGGCCCACTACGTGAACCATCACCC
TAATCAAGTTTTTTGGGGTCGAGGTGCCGTAAAGCACTAAATCGGAACCCTAAAGGGAGCCCCCG
ATTTAGAGCTTGACGGGGAAAGCCGGCGAACGTGGCGAGAAAGGAAGGGAAGAAAGCGAAAGG
AGCGGGCGCTAGGGCGCTGGCAAGTGTAGCGGTCACGCTGCGCGTAACCACCACACCCGCCGCGC
TTAATGCGCCGCTACAGGGCGCGTCCATTCGCCATTCAGGCTGCGCAACTGTTGGGAAGGGCGATC
GGTGCGGGCCTCTTCGCTATTACGCCAGCTGGCGAAAGGGGGATGTGCTGCAAGGCGATTAAGTT
GGGTAACGCCAGGGTTTTCCCAGTCACGACGTTGTAAAACGACGGCCAGTGAATTGTAATACGAC
TCACTATAGGGCGAATTGGGCCCGACGTCGCATGCTCCCGGCCGCCATGGCGGCCGCGGGAATTC
GATTAGTCAAGGACTAGTGATAAGTGGATGCCATCAGGTTTCCCGAGCGCTGCGGCCAGTTCATTC
AGCGTATAATCACTAGTGAATTCGCGGCCGCCTGCAGGTCGACC

3.5 kbp Substrate

60 nt Oligo

CTGTTCATTATCATCGCTTTTAAAACGGTTCGACCTTCTAATCCTATCTGACCATTATAA

3.5 kbp

GCCCAAGCCATTAATGGATCATTTTTCCATTTTTCAATAACATTATTGTTATACCAAATGTCATATCCTATAATCT
GGTTTTTGTTTTTTTGAATAATAAATGTTACTGTTCTTGCGGTTTGGAGGAATTGATTCAAATTCAAGCGAAAT
AATTCAGGGTCAAAATATGTATCAATGCAGCATTTGAGCAAGTGCGATAAATCTTTAAGTCTTCTTTCCCATGG
TTTTTTAGTCATAAAACTCTCCATTTTGATAGGTTGCATGCTAGATGCTGATATATTTTAGAGGTGATAAAATTA
ACTGCTTAACTGTCAATGTAATACAAGTTGTTTGATCTTTGCAATGATTCTTATCAGAAACCATATAGTAAATTA
GTTACACAGGAAATTTTTAATATTATTATTATCATTCATTATGTATTAAAATTAGAGTTGTGGCTTGGCTCTGCTA
ACACGTTGCTCATAGGAGATATGGTAGAGCCGCAGACACGTCGTATGCAGGAACGTGCTGCGGCTGGCTGGT
GAACTTCCGATAGTGCGGGTGTTGAATGATTTCCAGTTGCTACCGATTTTACATATTTTTTGCATGAGAGAATT
TGTACCACCTCCCACCGACCATCTATGACTGTACGCCACTGTCCCTAGGACTGCTATGTGCCGGAGCGGACAT
TACAAACGTCCTTCTCGGTGCATGCCACTGTTGCCAATGACCTGCCTAGGAATTGGTTAGCAAGTTACTACCG
GATTTTGTAAAAACAGCCCTCCTCATATAAAAAGTATTCGTTCACTTCCGATAAGCGTCGTAATTTTCTATCTT
TCATCATATTCTAGATCCCTCTGAAAAAATCTTCCGAGTTTGCTAGGCACTGATACATAACTCTTTTCCAATAA
TTGGGGAAGTCATTCAAATCTATAATAGGTTTCAGATTTGCTTCAATAAATTCTGACTGTAGCTGCTGAAACGT
TGCGGTTGAACTATATTTCCTTATAACTTTTACGAAAGAGTTTCTTTGAGTAATCACTTCACTCAAGTGCTTCC
CTGCCTCCAAACGATACCTGTTAGCAATATTTAATAGCTTGAAATGATGAAGAGCTCTGTGTTTGTCTTCCTGC
CTCCAGTTCGCCGGGCATTCAACATAAAAACTGATAGCACCCGGAGTTCCGGAAACGAAATTTGCATATACCC
ATTGCTCACGAAAAAAAATGTCCTTGTCGATATAGGGATGAATCGCTTGGTGTACCTCATCTACTGCGAAAACT
TGACCTTTCTCTCCCATATTGCAGTCGCGGCACGATGGAACTAAATTAATAGGCATCACCGAAAATTCAGGATA
ATGTGCAATAGGAAGAAAATGATCTATATTTTTTGTCTGTCCTATATCACCACAAAATGGACATTTTTCACCTGA
TGAAACAAGCATGTCATCGTAATATGTTCTAGCGGGTTTGTTTTTATCTCGGAGATTATTTTCATAAAGCTTTTCT
AATTTAACCTTTGTCAGGTTACCAACTACTAAGGTTGTAGGCTCAAGAGGGTGTGTCCTGTCGTAGGTAAATAA
CTGACCTGTCGAGCTTAATATTCTATATTGTTGTTCTTTCTGCAAAAAAGTGGGGAAGTGAGTAATGAAATTATT
TCTAACATTTATCTGCATCATACCTTCCGAGCATTTATTAAGCATTTCGCTATAAGTTCTCGCTGGAAGAGGTAGT
TTTTTCATTGTACTTTACCTTCATCTCTGTTCATTATCATCGCTTTTAAAACGGTTCGACCTTCTAATCCTATCTGA
CCATTATAATTTTTTAGAATGGTTTCATAAGAAAGCTCTGAATCAACGGACTGCGATAATAAGTGGTGGTATCCA
GAATTTGTCACTTCAAGTAAAAACACCTCACGAGTTAAAACACCTAAGTTCTCACCGAATGTCTCAATATCCGG
ACGGATAATATTTATTGCTTCTCTTGACCGTAGGACTTTCCACATGCAGGATTTTGGAACCTCTTGCAGTACTACT
GGGGAATGAGTTGCAATTATTGCTACACCATTGCGTGCATCGAGTAAGTCGCTTAATGTTCGTAAAAAAGCAGA
GAGCAAAGGTGGATGCAGATGAACCTCTGGTTCATCGAATAAAACTAATGACTTTTCGCCAACGACATCTACTA
ATCTTGTGATAGTAAATAAAACAATTGCATGTCCAGAGCTCATTCGAAGCAGATATTTCTGGATATTGTCATAAAA
CAATTTAGTGAATTTATCATCGTCCACTTGAATCTGTGGTTCATTACGTCTTAACTCTTCATATTTAGAAATGAGGC
TGATGAGTTCCATATTTGAAAAGTTTTCATCACTACTTAGTTTTTTGATAGCTTCAAGCCAGAGTTGTCTTTTTCT
ATCTACTCTCATACAACCAATAAATGCTGAAATGAATTCTAAGCGGAGATCGCCTAGTGATTTTAAACTATTGCT
GGCAGCATTCTTGAGTCCAATATAAAAGTATTGTGTACCTTTTGCTGGGTCAGGTTGTTCTTTAGGAGGAGTAA
AAGGATCAAATGCACTAAACGAAACTGAAACAAGCGATCGAAAATATCCCTTTGGGATTCTTGACTCGATAAG
TCTATTATTTTCAGAGAAAAAATATTCATTGTTTTCTGGGTTGGTGATTGCACCAATCATTCCATTCAAAATTGTT
GTTTTACCACACCCATTCCGCCCGATAAAAGCATGAATGTTCGTGCTGGGCATAGAATTAACCGTCACCTCAAA
AGGTATAGTTAAATCACTGAATCCGGGAGCACTTTTTCTATTAAATGAAAAGTGGAAATCTGACAATTCTGGCA
AACCATTTAACACACGTGCGAACTGTCCATGAATTTCTGAAAGAGTTACCCCTCTAAGTAATGAGGTGTTAAGG
ACGCTTTCATTTTCAATGTCGGCTAATCGATTTGGCCATACTACTAAATCCTGAATAGCTTTAAGAAGGTTATGTT
TAAAACCATCGCTTAATTTGCTGAGATTAACATAGTAGTCAATGCTTTCACCTAAGGAAAAAAACATTTCAGGGA
GTTGACTGAATTTTTTATCTATTAATGAATAAGTGCTTACTTCTTCTTTTTGACCTACAAAACCAATTTTAACATTTC
CGATATCGCATTTTTCACCATGCTCATCAAAGACAGTAAGATAAAACATTGTAACAAAGGAATAGTCATTCCAAC
CATCTGCTCGTAGGAATGCCTTATTTTTTTCTACTGCAGGAATATACCCGCCTCTTTCAATAACACTAAACTCCAA
CATATAGTAACCCTTAATTTTATTAAAATAACCGCAATTTATTTGGCGGCAACACAGGATCTCTCTTTTAAGTTACT
CTCTATTACATACGTTTTCCATCTAAAAATTAGTAGTATTGAACTTAACGGGGCATCGTATTGTAGTTTTCCATATTT
AGCTTTCTGCTTCCTTTTGGATAACCCACTGTTATTCATGTTGC
